# Supplementary material for: Perioperative Neurocognitive Disorder in Individuals with a History of Traumatic Brain Injury: Protocol for a Systematic Review and Meta-Analysis
Source: Biology (Basel). 2025 Feb 13;14(2):197. doi: 10.3390/biology14020197 (PMC11852134; doi:10.3390/biology14020197)
Supplement: Supplementary file 1 [file biology-14-00197-s001.zip › biology-3359196-supplementary.pdf]

Supplementary File S1: Search string used to identify relevant articles from various databases.

Database: Ovid MEDLINE(R) and In-Process, In-Data-Review & Other Non-Indexed Citations <1946 to February 20, 2024>

1 exp Brain Injuries, Traumatic/ 25558

2 Traumatic brain injury.ti,ab,kf. 48261

3 ((head or crani* or cerebr* or capitis or brain* or forebrain* or skull* or hemispher* or intra?cran* or inter?cran* or intracran* or intercran*) adj3 (injur* or trauma* or damag* or lesion* or wound* or destruction* or contusion* or concus* or fracture*)).ti,ab. 225160

4 exp Craniocerebral Trauma/ 181601

5 Craniocerebral Trauma.ti,ab,kf. 1628

6 exp Brain Concussion/ 12714

7 1 or 2 or 3 or 4 or 5 or 6 323650

8 exp Decompressive Craniectomy/ 1883

9 haematoma evacuation.mp. 128

10 Endoscopic Ventriculostomy.mp. 58

11 exp Ventriculostomy/ 2807

12 exp Ventriculoperitoneal Shunt/ 4997

13 Cranioplasty.mp. 3096

14 Extracranial surgery.ti,ab,kf. 33

15 8 or 9 or 10 or 11 or 12 or 13 or 14 11998

16 exp Dexmedetomidine/ 5612

17 exp Propofol/ 17073

18 exp Ketamine/ 15310

19 exp Thiopental/ 6899

20 exp Etomidate/ 2084

21 exp Sevoflurane/ 7280

22 exp Isoflurane/ 9823

23 exp Desflurane/ 1774

24 exp Midazolam/ 9920

25 exp Fentanyl/ 18041

26 exp Rocuronium/ 2191

27 exp Sufentanil/ 2186

28 exp Vecuronium/ 2139

29 exp Morphine/ 40711

30 (Dexmedetomidine or Propofol or Ketamine or Thiopental or Etomidate or Sevoflurane or Isoflurane or Desflurane or Midazolam or Ketofol or Fentanyl or Rocuronium or Sufentanil or Vecuronium or Morphine).ti,ab. 151288

31 16 or 17 or 18 or 19 or 20 or 21 or 22 or 23 or 24 or 25 or 26 or 27 or 28 or 29 or 30 170937

32 15 or 31 182874

33 7 and 32 4951

34 exp cohort studies/ 2569735

35 cohort$.tw. 892942

36 controlled clinical trial.pt. 95543

37 epidemiologic methods/ 31621

38 limit 37 to yr=1966-1989 11199

39 exp case-control studies/ 1479575

40 (case$ and control$).tw. 617518

41 (case$ and series).tw. 237069

42 case reports.pt. 2381603

43 (case$ adj2 report$).tw. 716992

44 (case$ adj2 stud$).tw.321302

45 34 or 35 or 36 3032917

46 38 or 39 or 40 or 41 or 42 or 43 or 44 4590629

47 45 or 46 6204721

48 33 and 47 2245

49 limit 48 to english language **2041**

Link:

https://ezproxy.library.yorku.ca/login?url=http://ovidsp.ovid.com/ovidweb.cgi?T=JS&NEWS=N&PAGE=ma
in&SHAREDSEARCHID=45lkg7sxHVcZFU3dWHxPqJaISQlNKvfII57mW0DhSnk3KgGlzwXQxoWNyLRPf
OySb (accessed on 12 January 2025).

Database: Embase <1974 to 2024 February 19>

1 exp traumatic brain injury/ 69365

2 'traumatic brain injury'.ti,ab. 66759

3 ((head or crani* or cerebr* or capitis or brain* or forebrain* or skull* or hemispher* or intra?cran* or inter?cran* or intracran* or intercran*) adj3 (injur* or trauma* or damag* or lesion* or wound* or destruction* or contusion* or concus* or fracture*)).ti,ab. 304749

4 exp Craniocerebral Trauma/ 347723

5 'Craniocerebral Trauma'.ti,ab. 1615

6 exp Brain Concussion/ 7454

7 1 or 2 or 3 or 4 or 5 or 6 472804

8 exp Decompressive Craniectomy/ 6370

9 'haematoma evacuation'.ti,ab. 196

10 'Endoscopic Ventriculostomy'.ti,ab. 85

11 exp Ventriculostomy/ 3039

12 exp Ventriculoperitoneal Shunt/ 13648

13 Cranioplasty.ti,ab. 3598

14 'Extracranial surgery'.ti,ab. 46

15 8 or 9 or 10 or 11 or 12 or 13 or 14 24913

16 exp Dexmedetomidine/ 19898

17 exp Propofol/ 73633

18 exp Ketamine/ 62323

19 exp Thiopental/ 24962

20 exp Etomidate/ 9523

21 exp Sevoflurane/ 27878

22 exp Isoflurane/ 39284

23 exp Desflurane/ 7030

24 exp Midazolam/ 62668

25 exp Fentanyl/ 80417

26 exp Rocuronium/ 16063

27 exp Sufentanil/ 12510

28 exp Vecuronium/ 12673

29 exp Morphine/ 117216

30 (Dexmedetomidine or Propofol or Ketamine or Thiopental or Etomidate or Sevoflurane or Isoflurane or Desflurane or Midazolam or Ketofol or Fentanyl or Rocuronium or Sufentanil or Vecuronium or Morphine).ti,ab. 209544

31 16 or 17 or 18 or 19 or 20 or 21 or 22 or 23 or 24 or 25 or 26 or 27 or 28 or 29 or 30 376196

32 15 or 31 400474

33 7 and 32 14803

34 exp cohort analysis/ 1115202

35 exp longitudinal study/ 206597

36 exp prospective study/ 905150

37 exp follow up/ 2141862

38 cohort$.tw. 1533363

39 exp case control study/ 231533

40 (case$ and control$).tw. 914384

41 exp case study/ 99518

42 (case$ and series).tw.345121

43 case report/ 2967065

44 (case$ adj2 report$).tw. 966768

45 (case$ adj2 stud$).tw.429554

46 34 or 35 or 36 or 37 or 38 or 39 or 40 or 41 or 42 or 43 or 44 or 45 7859892

47 33 and 46 5970

48 limit 47 to english language **5707**

Embase cohort, case-control, case series, and case study strategy Filters[1]

Link:

https://ezproxy.library.yorku.ca/login?url=http://ovidsp.ovid.com/ovidweb.cgi?T=JS&NEWS=N&PAGE=main&SHAREDSEARCHID=7RZ57MNCox5jvdhjtR1HAT6PmRglfafqOl9t0XWhTsMCaUEM0Sv591BYYhwGl0O07 (accessed on 12 January 2025)

[1] https://bestpractice.bmj.com/info/toolkit/learn-ebm/study-design-search-filters/

Database: Ovid Emcare <1995 to 2024 Week 07>

1 exp traumatic brain injury/ 16342

2 'traumatic brain injury'.ti,ab. 20708

3 ((head or crani* or cerebr* or capitis or brain* or forebrain* or skull* or hemispher* or intra?cran* or inter?cran* or intracran* or intercran*) adj3 (injur* or trauma* or damag* or lesion* or wound* or destruction* or contusion* or concus* or fracture*)).ti,ab. 72433

4 exp Craniocerebral Trauma/ 61625

5 'Craniocerebral Trauma'.ti,ab. 204

6 exp Brain Concussion/ 1056

7 1 or 2 or 3 or 4 or 5 or 6 99456

8 exp Decompressive Craniectomy/ 696

9 'haematoma evacuation'.ti,ab. 43

10 'Endoscopic Ventriculostomy'.ti,ab. 6

11 exp Ventriculostomy/ 212

12 exp Ventriculoperitoneal Shunt/ 1701

13 Cranioplasty.ti,ab. 446

14 'Extracranial surgery'.ti,ab. 9

15 8 or 9 or 10 or 11 or 12 or 13 or 14 2964

16 exp Dexmedetomidine/ 3748

17 exp Propofol/ 22321

18 exp Ketamine/ 9327

19 exp Thiopental/ 5596

20 exp Etomidate/ 2607

21 exp Sevoflurane/ 9927

22 exp Isoflurane/ 8372

23 exp Desflurane/ 2947

24 exp Midazolam/ 15700

25 exp Fentanyl/ 22926

26 exp Rocuronium/ 5376

27 exp Sufentanil/ 3799

28 exp Vecuronium/ 4947

29 exp Morphine/ 22084

30 (Dexmedetomidine or Propofol or Ketamine or Thiopental or Etomidate or Sevoflurane or Isoflurane or Desflurane or Midazolam or Ketofol or Fentanyl or Rocuronium or Sufentanil or Vecuronium or Morphine).ti,ab. 57742

31 16 or 17 or 18 or 19 or 20 or 21 or 22 or 23 or 24 or 25 or 26 or 27 or 28 or 29 or 30 92683

32 15 or 31 95500

33 7 and 32 **2771**

Link:

https://ezproxy.library.yorku.ca/login?url=http://ovidsp.ovid.com/ovidweb.cgi?T=JS&NEWS=N&PAGE=main&SHAREDSEARCHID=3RBDkezOuHiyhjzDrER5aRl9rI1yFibCJPOXuT0rPVxAZp5CxVsKAUwgVIbf1Akzo (accessed on 12 January 2025)

Database: APA PsycInfo <1806 to February Week 3 2024>

1 exp traumatic brain injury/ 23766

2 'traumatic brain injury'.ti,ab. 19831

3 ((head or crani* or cerebr* or capitis or brain* or forebrain* or skull* or hemispher* or intra?cran* or inter?cran* or intracran* or intercran*) adj3 (injur* or trauma* or damag* or lesion* or wound* or destruction* or contusion* or concus* or fracture*)).ti,ab. 63428

4 exp Craniocerebral Trauma/ 8094

5 'Craniocerebral Trauma'.ti,ab. 87

6 exp Brain Concussion/ 3538

7 1 or 2 or 3 or 4 or 5 or 6 66160

8 exp Decompressive Craniectomy/ 0

9 'haematoma evacuation'.ti,ab. 4

10 'Endoscopic Ventriculostomy'.ti,ab. 2

11 exp Ventriculostomy/ 0

12 exp Ventriculoperitoneal Shunt/ 0

13 Cranioplasty.ti,ab. 69

14 'Extracranial surgery'.ti,ab. 2

15 8 or 9 or 10 or 11 or 12 or 13 or 14 77

16 exp Dexmedetomidine/ 0

17 exp Propofol/ 623

18 exp Ketamine/ 3106

19 exp Thiopental/ 72

20 exp Etomidate/ 0

21 exp Sevoflurane/ 0

22 exp Isoflurane/ 0

23 exp Desflurane/ 0

24 exp Midazolam/ 571

25 exp Fentanyl/ 868

26 exp Rocuronium/ 0

27 exp Sufentanil/ 0

28 exp Vecuronium/ 0

29 exp Morphine/ 7332

30 (Dexmedetomidine or Propofol or Ketamine or Thiopental or Etomidate or Sevoflurane or Isoflurane or Desflurane or Midazolam or Ketofol or Fentanyl or Rocuronium or Sufentanil or Vecuronium or Morphine).ti,ab. 20123

31 16 or 17 or 18 or 19 or 20 or 21 or 22 or 23 or 24 or 25 or 26 or 27 or 28 or 29 or 30 20595

32 15 or 31 20672

33 7 and 32 321

Link:

https://ezproxy.library.yorku.ca/login?url=http://ovidsp.ovid.com/ovidweb.cgi?T=JS&NEWS=N&PAGE=main&SHAREDSEARCHID=1LP2zkaLKMJd8wT1TR11FE5KqwHIZ9Rv002P19fiFgNasauXkG9XvqBMigL304prF (accessed on 12 January 2025)
